# Improved induced innate immune response after cART initiation in people with HIV

**DOI:** 10.3389/fimmu.2022.974767

**Published:** 2022-08-17

**Authors:** Malene Hove-Skovsgaard, Dina Leth Møller, Annemette Hald, Jan Gerstoft, Jens Lundgren, Sisse Rye Ostrowski, Susanne Dam Nielsen

**Affiliations:** ^1^ Department of Infectious Diseases, Rigshospitalet, Copenhagen University Hospital, Copenhagen, Denmark; ^2^ Department of Clinical Medicine, University of Copenhagen, Copenhagen, Denmark; ^3^ Centre of Excellence for Health, Immunity, and Infections, Department of Infectious Diseases, Rigshospitalet, Copenhagen University Hospital, Copenhagen, Denmark; ^4^ Department of Clinical Immunology, Rigshospitalet, Copenhagen University Hospital, Copenhagen, Denmark

**Keywords:** Human immunodeficiency virus (HIV), innate immune response, cytokines, TruCulture, lipopolysaccharide, resiquimod, polyinosinic:polycytidylic acid, toll-like receptor

## Abstract

**Introduction:**

Impairment of the innate immune function may contribute to the increased risk of bacterial and viral infections in people with HIV (PWH). In this study we aimed to investigate the induced innate immune responses in PWH prior to and after initiation of combinational antiretroviral therapy (cART). Furthermore, we aimed to investigate if the induced innate immune responses before initiation of cART were associated with CD4+ T-cell recovery one year after initiating cART.

**Material and method:**

The induced innate immune response was assessed by the TruCulture^®^ whole blood technique in 32 PWH before cART initiation and after 1, 6 and 12 months. To mimic bacterial and viral infections we used a panel of three stimuli (lipopolysaccharide (LPS), resiquimod (R848), and polyinosinic:polycytidylic acid (Poly I:C)) to stimulate the extracellular Toll-like receptor (TLR) 4 and the intracellular TLR7/8 and TLR3, respectively. The following cytokine responses were analyzed by Luminex 200: Tumor Necrosis Factor (TNF)-α, Interleukin (IL)-1b, IL-6, IL-8, IL-10, IL-12p40, IL17A, Interferon (IFN)-α, and IFN-γ.

**Results:**

At baseline PWH with nadir CD4+ T-cell count <350 cell/µL had lower levels of LPS-, R848-, and Poly I:C-induced IL-6 and IFN-γ, LPS- and R848-induced TNF-α and IL-12, LPS induced IL-1b, and R848-induced IL-10 than PWH with nadir CD4+ T-cell count >350 cells/µL. The majority (>50%) had induced cytokine concentrations below the reference intervals at baseline which was most pronounced for the LPS- and Poly I:C-induced responses. The induced responses in the whole population improved after 12 months of cART, and more PWH had induced cytokine concentrations within the reference intervals after 12 months. However, the majority of PWH still had LPS-induced INF-α, INF-γ and Poly I:C-induced TNF-α and IL-6 below the reference interval. The induced innate immune responses before cART initiation were not associated with the CD4+ T-cell recovery after 12 months of cART.

**Conclusion:**

The innate immune response was impaired in PWH, with a more pronounced impairment in PWH with low nadir CD4+ T-cell count. Initiation of cART improved the innate immune response, but compared to the reference intervals, some impairment remained in PWH without viral replication.

## Introduction

Infection with human immunodeficiency virus (HIV) causes substantial immune deficiency with a progressive loss of CD4+ T-cells and an increased susceptibility to infections (1). Combination antiretroviral therapy (cART) leads to restoration of CD4+ T-cells, prevents the development of acquired immune deficiency syndrome (AIDS), and has dramatically decreased morbidity and mortality in people with HIV (PWH) (1–3). However, even though cART substantially reduces the incidence of opportunistic infections, the risk of developing bacterial and viral infections, as well as malignancies including those related to pro-oncogenic viruses, are still elevated in PWH in the cART era (4–9). For some pathogens, a common route of infection with HIV may account for a part of the risk, but residual immune dysfunction is thought to play a central role.

Although the main target for HIV is CD4+ T-cells, other CD4+ positive cells including innate immune cells such as monocytes, macrophages, and dendritic cells are susceptible to HIV infection, and a more comprehensive impact on the immune system has been described (1). This includes increased innate immune activation due to both direct effects of HIV infection and indirect effects such as microbial translocation from the gut (10, 11). Initiation of cART has been reported to reduce, but not normalize, innate immune activation and partly restore innate cell count and phenotypes (12–14). However, the functional innate immune response is less studied. Most studies investigating the functional innate immune response in PWH are limited by their cross-sectional design, and results have been conflicting with reports of both increased and decreased functional responses to various pathogens compared to the uninfected population (15–21). In addition, most studies were conducted on specific cell subsets, thus not investigating the response of all immunologically active circulating cells. This includes the few smaller prospective studies that have been conducted to investigate the impact of cART initiation on the functional innate immune response (22–24). Therefore, we conducted this prospective study using a commercially available whole blood assay to obtain a broad and comprehensive profiling of the innate immune responses to ligands stimulating both intracellular and extracellular Toll-like receptor (TLR) signaling pathways, mimicking both bacterial and viral infections. Use of a standardized commercially available assay makes the study easily reproducible and enables comparison between laboratories. TLRs recognize distinct molecular structures that are shared by pathogens, and the receptors are essential for the initiation of an innate immune response. When a ligand is recognized by a TLR this initiates downstream signaling events that lead to the secretion of inflammatory cytokines, type I Interferons, chemokines, and antimicrobial peptides which further recruit and activate innate immune cells in the defense against the pathogen (25).

We aimed to investigate the induced innate immune responses in PWH prior to and after initiation of cART. Furthermore, we aimed to investigate if the induced innate immune responses before initiation of cART were associated with CD4+ T-cell recovery one year after initiating cART.

## Materials and methods

### Study population

A total of 32 PWH were included in this prospective cohort study when initiating cART at The Department of Infectious Diseases at Rigshospitalet, Copenhagen University Hospital, Denmark, and followed for 12 months. Inclusion criteria were confirmed HIV diagnoses, naïve to cART, and no other immunodeficiency or immunosuppressive treatment at the time of diagnosis. We did not have any exclusion criteria. Due to the laboratory setup, inclusion was only possible within normal working hours between Monday to Thursday. Thus, the included patients represent a convenience sample. All participants contributed with a blood sample at baseline, before initiating cART, and were invited to participate with blood samples 1, 6, and 12 months after cART initiation. Patient characteristics and HIV specific data were obtained from medical records and included age, sex, cART, nadir CD4+ T-cell count, current CD4+ and CD8+ T-cell count, HIV RNA, time of HIV diagnosis, and hepatitis B and C serostatus.

### TruCulture^®^ analyses

The TruCulture^®^ assay was developed as a standardized method to measure immune responses (26). TruCulture^®^ tubes (Myriad RBM, Austin, TX, USA) were pre-coated by the manufacturer and stored at -20° C until use. Three different stimuli were used, including lipopolysaccharide (LPS) from Escherichia coli O111:B4 known to stimulate the TLR4 pathway (cat.# 782-001261), resiquimod (R848), a synthetic agonist mimicking single-stranded RNA known to stimulate the TLR7/8 pathway (cat.#782-001264), and polyinosinic:polycytidylic acid (Poly I:C) an analogue of double-stranded RNA known to stimulate the TLR3 pathway (cat.# 782-001282). Furthermore, a control tube only containing TruCulture^®^ medium (NULL) was included. The tubes were custom-made with reduced heparin since the blood was collected in heparin containing tubes and transferred to the TruCulture^®^ tubes. The whole blood was stimulated for 22 hours (+/- 30 min) at 37° C in a digital dry block heater (VWR International A/S Søborg, Denmark) according to the manufacturer’s instructions. The supernatants were harvested and stored at -80°C until use.

### Cytokine analyses

Supernatants from the TruCulture^®^ stimulations were thawed, and the concentrations of nine cytokines were measured: Tumor Necrosis Factor (TNF)-α, Interleukin (IL)-1b, IL-6, IL-8, IL-10, IL-12p40, IL17A, Interferon (IFN)-α, and IFN-γ. Cytokine concentrations were determined by Luminex assay (R&D Systems, BIO-Techne LTD, Minneapolis, MN, USA) using a Luminex 200 instrument (R&D Systems) according to the manufacturers protocol. In short, 50 μL of microparticle cocktail and 50 μL sample were added to each well in the microplate and incubated for 2 hours at room temperature. The microplate was washed three times and 50 μL of diluted biotin antibody cocktail was added to each well and incubated for one hour. Afterwards, the microplate was washed three times and 50 μL of diluted streptavidin-PE was added to each well and incubated for 30 minutes and washed three times. The microparticles were resuspended in 100 μL wash buffer, incubated in 2 minutes, and the cytokine concentrations were analyzed. All cytokine concentrations were measured in pg/mL. Reference intervals for each cytokine in all stimulated and unstimulated samples were in-house references provided by the Department of Clinical Immunology based on the 5-95% interval.

### Statistical analyses

Continuous data were presented as median and interquartile range (IQR) and proportion as percentages. To compare stimulated cytokine responses at baseline in participants with nadir CD4+ T-cell count below and above 350 cells/µL, we used the Mann-Whitney U test. To analyze changes in induced cytokine responses over time, we applied linear mixed models including follow-up time (categorical) as a fixed effect. To account for the correlation in the repeated measurements as well as possible variance heterogeneity over time, we assumed an unstructured covariance pattern (R packages: LMM star) (27). Cytokine concentrations were log-10 transformed and values below detection level were set to 1% of the lowest value for the given cytokine to enable transformation. Percentages for participants with induced cytokines concentration below and above the reference interval (5-95%) were given for participants with samples at baseline and 12 months follow-up. All correlations were based on Spearman’s rank correlation coefficients. The correlation matrices included participants with samples from baseline and 12 months follow-up (R packages: corrplot). For cluster analyses, baseline log-10 transformed induced cytokine concentrations were scaled before the generation of dendrograms and heatmaps. IFN-α was not included in the analyses due to missing data in the baseline samples. Generation of dendrograms were drawn based on hierarchical clustering analysis using the complete agglomeration method on Euclidian distance matrices (R packages: gplot). All analyses were performed in R version 3.6.1.

## Results

### Study population

Clinical characteristics of the 32 participants are shown in **Table 1**. Most participants were male (97%) with a median age of 42 years at inclusion. None of the participants were co-infected with hepatitis B, and only 1 (3.1%) participant was co-infected with hepatitis C. Eight (25%) of the participants had AIDS when diagnosed with HIV and initiating cART. At baseline the median CD4+ T-cell count was 315 (198-439) cells/µL which was equivalent to the nadir CD4+ T-cell count in this study population. After one year of cART, the median CD4+ T-cell count was 515 (350-760) cells/µL. All the participants achieved viral suppression within the first year of treatment (HIV RNA < 50 copies/mL). All participants contributed with blood samples before cART initiation. For participants contributing with follow-up samples, please see **Table 1**.

**Table 1:** Clinical characteristics.

Clinical Characteristics	n
Participants (n)	32
Baseline sample (n)	32
1-month sample (n)	30
6-months sample (n)	18
12 months sample (n)	23
Age (years), median (IQR)	42 (30-52)
Sex (male), n (%)	31 (97)
Baseline/nadir CD4+ T-cell (cells/µL), median (IQR)	315 (198-439)
Baseline CD8+ T-cell (cells/µL), median (IQR)	760 (657-970)
Baseline HIV RNA (copies/mL), median (IQR)	82.9x10^3^ (25.4x10^3^-201.5 x10^3^)
CD4+ T-cell after 1-year of cART (cells/µL), median (IQR)	515 (350-760)
CD8+ T-cell after 1-year of cART CD8+ (cells/µL), median (IQR)	785 (673-970)
HIV RNA after 1-year of cART (copies/mL), median (IQR)	<20 (20-28)
Hepatitis C, positive, n (%)	1 (3.1)
Hepatitis B, positive, n (%)	0 (0)
Time since HIV diagnosis (days), median (IQR)	7 (4-14)
AIDS-defining disease at inclusion, n (%)	8 (25)
CD4+ < 350 cells/µL at inclusion, n (%)	18 (56)
CD4+ > 500 cells/µL at inclusion, n (%)	7 (22)

### Induced cytokine responses before cART initiation

**Figure 1:**
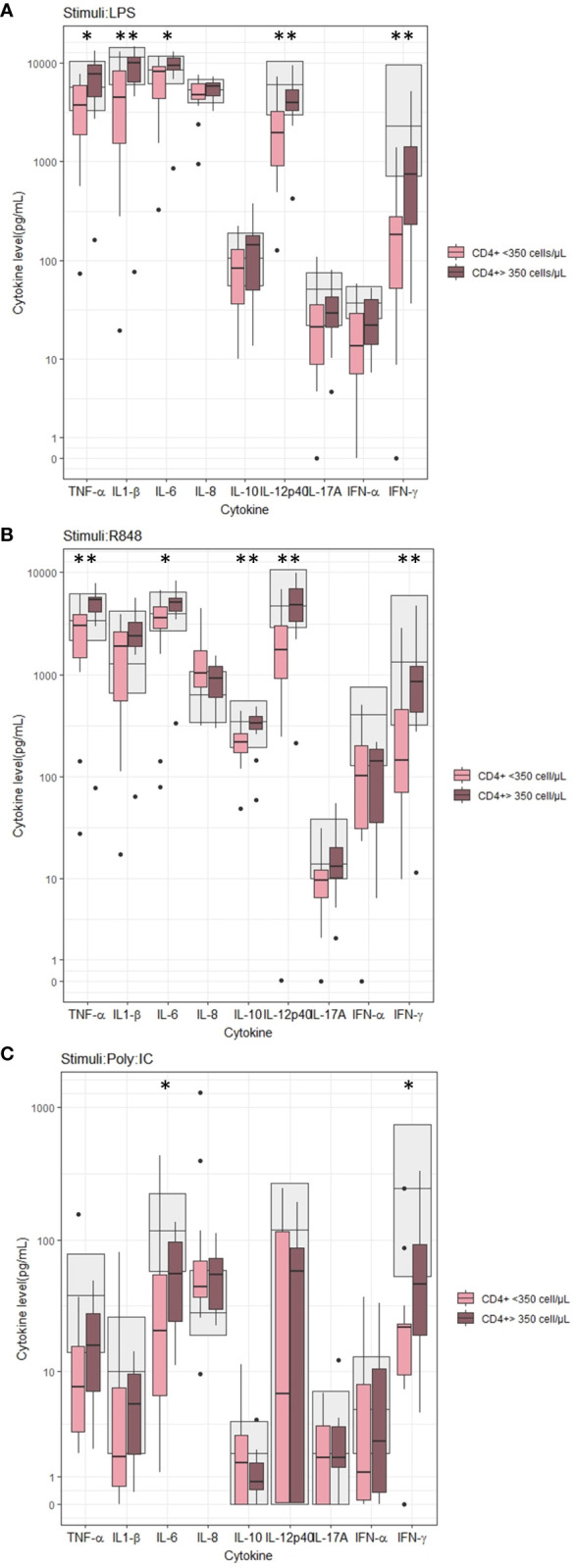
Induced cytokine concentration according to nadir CD4+ T-cell count before cART initiation. **(A)** Stimuli: LPS, **(B)** Stimuli: R848, **(C)** Stimuli: PolyI:C. Grey areas indicates 5-95% reference interval. * and ** indicates p-values ≤ 0.05 and ≤ 0.01, respectively.

At baseline, we found lower LPS-induced TNF-α (p=0.014), IL-1β (p=0.010), IL-6 (p=0.025), IL-12p40 (p=0.006), and IFN-γ (p=0.006) concentrations in PWH with nadir CD4+ T-cells < 350 cells/µL than in PWH with CD4+ T-cell count > 350 cells/µL (**Figure 1A**). Similarly, the R848-induced TNF-α (p=0.002), IL-6 (p=0.025), IL-10 (p=0.008), IL-12p40 (p=0.002), and IFN-γ (p=0.002) concentrations (**Figure 1B**) and Poly I:C-induced IL-6 (p=0.037) and IFN-γ (p=0.035) concentrations (**Figure 1C**) were lower in PWH with nadir CD4+ T-cells < 350 cells/µL than in PWH with nadir CD4+ T-cell count > 350 cells/µL. No differences between the two groups were found in the unstimulated samples (data not shown).

### Development of cytokine responses within the first year of treatment with cART

**Figure 2:**
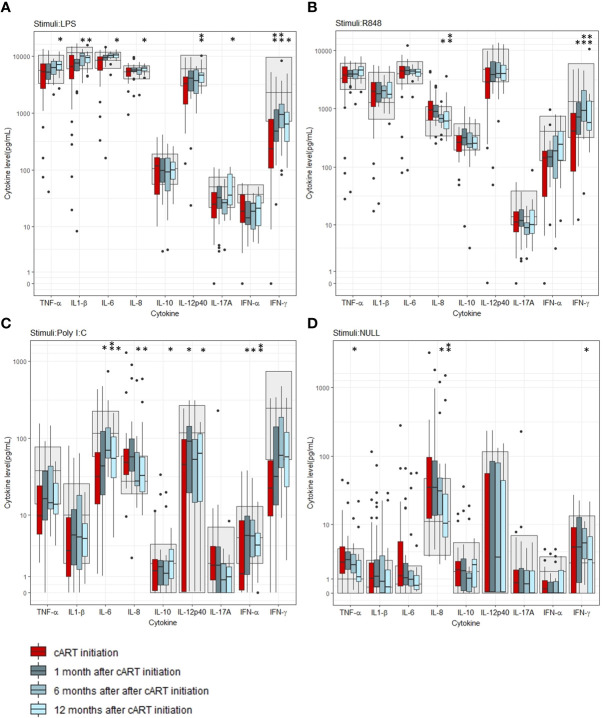
Induced cytokine concentrations before and after initiation of combination antiviral treatment (cART). **(A)** Stimuli: LPS, **(B)** Stimuli: R848, **(C)** Stimuli: Poly I:C, **(D)** Unstimulated (Null). Grey areas indicate 5-95% reference interval. * and ** indicates p-values < 0.05 and 0.005, respectively.

We measured the induced cytokine responses before cART initiation and after 1 month, 6 months and 12 months of treatment. The LPS-induced TNF-α (p=0.012), IL-1β (p=0.008), IL-6 (p=0.004), IL-8 (p=0.012), IL-12p40 (p=0.002), IL17A (p=0.029), and IFN-γ (p=0.006) concentrations all increased after 12 months of cART compared to before cART initiation (**Figure 2A**). Also, an increase in LPS-induced IFN-γ concentration after 1 and 6 months (p=0.002 and p=0.001, respectively) and in LPS-induced IL-1β (p=0.015) concentration 6 months after cART initiation was found (**Figure 2A**). Before cART initiation, the majority (> 50%) of the participants had LPS-induced IFN-α (68%), IFN-γ (73%), IL-12 (52%), and IL-17A (56%) concentrations below the reference interval. After 12 months of cART, the majority of PWH had LPS-induced IL-12 and IL-17A concentrations within the reference interval but not the LPS-induced IFN-α (65%) and IFN-γ (60%) concentrations (**Figure 2A**).

The R848-induced IFN-γ concentration increased after 1 month, 6 months and 12 months of cART compared to before cART initiation (p=0.007, p=0.004, and p=0.004, respectively) (**Figure 2B**), while R848-induced IL-8 concentration decreased after 6 months and 12 months of cART (p=0.006 and p= 0.002, respectively) (**Figure 2B**). Before cART initiation, the majority had R848-induced IFN-α (53%) and IFN-γ (52%) concentrations below the reference interval, but after 12 months of cART, the majority had R848-induced cytokine concentrations within the reference interval.

The Poly I:C-induced IL-6 and IFN-α concentrations increased after 1 month, 6 months and 12 months of cART (p=0.025, p=0.002, p=0.028 and p=0.033, p=0.050, p=0.017, respectively). Also, the Poly I:C-induced IL-10 and IL-12p40 concentrations increased 12 months and 1 month and 12 months after cART initiation, respectively (p= 0.028 and p=0.005, p=0.039, respectively) (**Figure 2C**). Like the R848-induced response, the Poly I:C induced IL-8 concentration decreased after 6 months and 12 months of cART (p=0.037 and p=0.012) (**Figure 2C**). The majority of PWH had Poly I:C-induced IL-6 (69%), IFN-α (53%), and IFN-γ (73%) concentrations below the reference interval before cART initiation. After 12 months of cART, the majority had Poly I:C-induced TNF-α (52%) and IL-6 (56%) concentrations below the reference intervals (**Figure 2C**). For percentages of all induced cytokine concentrations below and above the reference intervals, please see **Table S1** in the **supplementary material**.

In the unstimulated samples, an increase in the INF-γ concentration was found after 6 months but not after 12 months of cART (p=0.010). Furthermore, a decrease in the pro-inflammatory TNF-α concentration after 6 months and IL-8 concentration after 6 months and 12 months of cART were found (p=0.009,p=0.039 and 0.002 respectively), (**Figure 2D**). For p-values of all cytokine comparisons at all timepoints, please see **Table S2** in supplementary.

In an exploratory analysis, a positive correlation was found between the Poly I:C induced IL-10 concentration and the unstimulated IL-10 concentration 12 months after cART initiation and between the delta values between baseline and 12 months after cART initiation for the Poly I:C induced IL-10 concentration and the unstimulated IL-10 concentration (rho=0.95, p<0.001 and rho=0.88, p <0.001, respectively) (**S3 in supplementary**)

### Intercorrelation of the cytokine response and correlation to viral load

**Figure 3:**
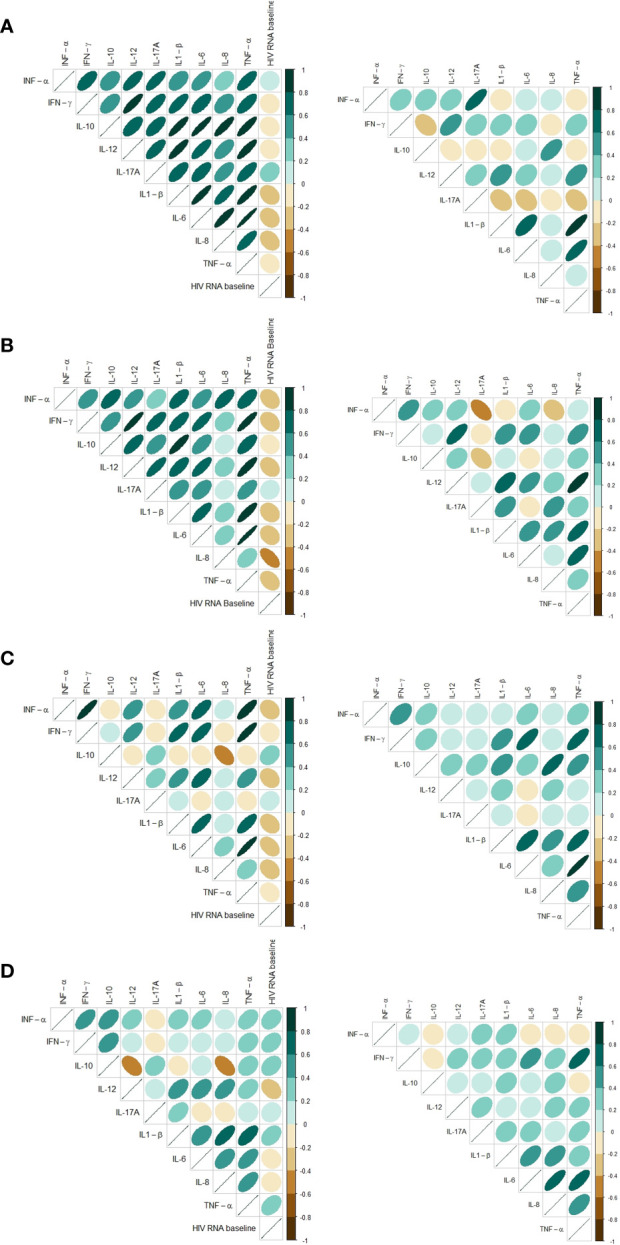
Correlation matrix based on Spearman’s rank correlation coefficients for baseline and 12 months follow-up showing cytokine intercorrelation and correlation to HIV RNA at baseline. Only includes participants with samples at both timepoints. **(A)** Stimuli: LPS, **(B)** Stimuli: R848, **(C)** Stimuli: Poly I:C, **(D)** Unstimulated (Null).

The intercorrelations between cytokines within each stimulus were different before and after cART. Before cART initiation the LPS- and R848-induced cytokine responses were all strongly, positively correlated. After 12 months of treatment with cART, the LPS- and R848-induced cytokine responses were less intercorrelated, and a few cytokines were negatively correlated (**Figures 3A, B**). This was not found in the Poly I:C-induced or unstimulated samples (**Figures 3C, D**).

Most induced cytokines had an inverse correlation to viral load at baseline in the LPS-, R848- and Poly I:C-induced samples (**Figures 3A–D**). In the unstimulated samples a tendency towards a positive correlation between cytokine concentrations and HIV RNA were found at baseline (**Figure 3D**). Since all participants had HIV RNA < 50 copies/mL after 12 months of cART the correlations were not investigated after 12 months.

### Pre-treatment cytokine signatures and CD4+ recovery after 12 months of cART

**Figure 4:**
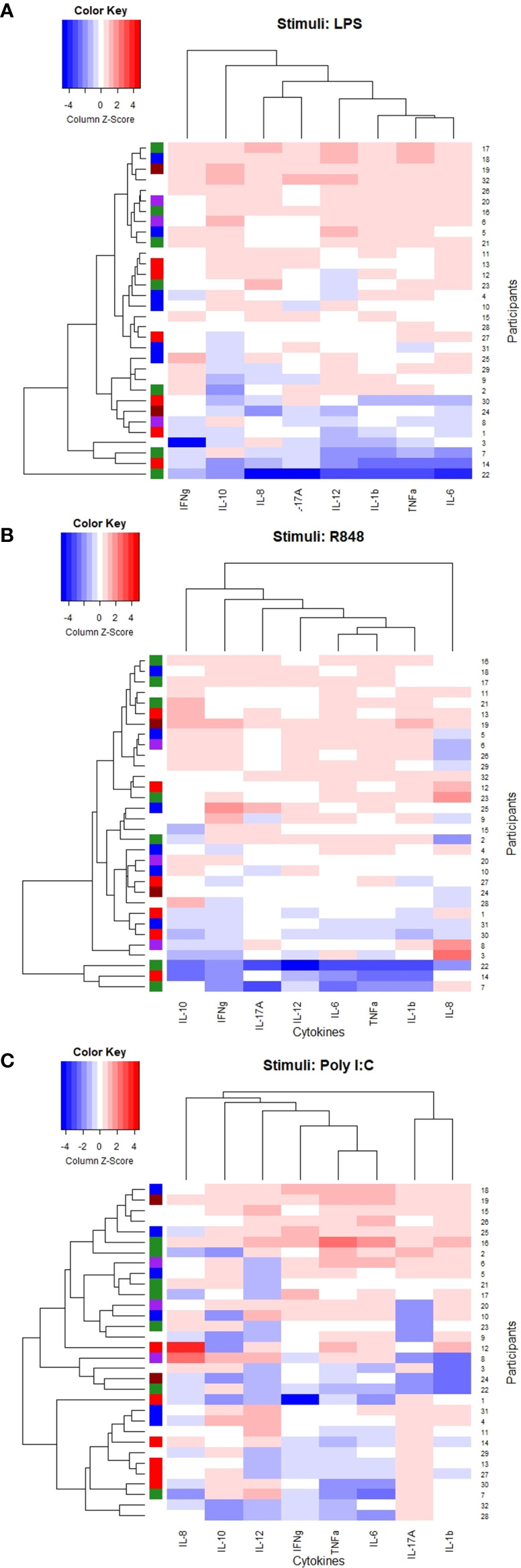
Heatmaps and dendrogram. Colored bar on the left side indicating delta CD4+ T-cell increase 12 months after initiating cART; brown <50 cells/µL, purple 50-99 cells/µL, green 100-199 cells/µL, red 200-300 cells/µL, blue >300 cells/µL, white: missing data. **(A)** Stimuli: LPS, **(B)** Stimuli: R848, **(C)** Stimuli: Poly I:C.

No distinct clusters reflected the delta CD4+ T-cell reconstruction after 12 months of cART since participants with delta CD4+ T-cell increase of <50, 50-100, 100-200 and > 200 cells/µL were mixed between all clusters in all induced responses (**Figures 4A–C**).

Both the LPS- and R848 induced TNF-α, IL-1b, IL-6, and IL-12p40 clustered together. Also, the Poly I:C-induced TNF-α, IL-6, and IL-12p40 clustered together but with the Poly I:C-induced IFN-γ and not the Poly I:C-induced IL-1b (**Figures 4A–C**).

As an exploratory analysis we correlated the baseline induced cytokine responses to delta CD4+ T-cell increase 12 months after cART initiation. Only the Poly I:C induced IL-17A concentration was significant correlated to delta CD4+ T-cell (rho= 0.399, p=0.044). For all correlations, please see **Table S4** in supplementary.

## Discussion

The innate immune function is essential in the primary defense against pathogens. To our knowledge this is the largest prospective study investigating the induced innate immune function in PWH and the impact of cART initiation. In this study, a nadir CD4+ T-cell count < 350 cells/µL in untreated PWH was associated with a lower induced innate immune response. Furthermore, we found a broad impairment of the induced cytokine response in PWH when stimulating both intra- and extracellular TLR receptors, compared to reference intervals, which improved after cART initiation. However, some innate induced cytokine responses remained below the reference interval after cART initiation, in line with the increased risk of infections in PWH. With the chosen unsupervised approach, we did not identify any clusters at baseline associated with the CD4+ immune recovery 12 months after initiating cART nor did the baseline induced cytokine concentration in general correlate with CD4+ immune recovery.

Previous studies on induced innate immune responses in PWH are conflicting. Some studies have found an impaired innate response to various pathogens in untreated PWH compared to PWH on cART (28) and in both untreated PWH and PWH on cART compared with uninfected controls (16, 17, 19, 22, 28, 29), but other studies found no differences in the induced response between PWH and uninfected controls (20, 30) or increased response in PWH compared with uninfected controls (21). However, previous studies have not had the option of using a commercial assay, and this may explain the variation due to differences in signaling pathways and cell types investigated, intracellular or extracellular measurements of cytokines and handling of the cells. We found a reduced induced immune response before cART in PWH with nadir CD4+ T-cell count < 350 cells/µL compared to participants with higher nadir CD4+ T-cell count. This was most pronounced for the LPS- and R848-induced responses. Low CD4+ T-cell counts often reflect a longer time since HIV infection and high chronic immune activation and have been associated with more severe gut dysbiosis, increased enterocyte damage and microbial translocation including elevated LPS in PWH (31–34). Thus, the lower LPS induced innate immune response in PWH with CD4+ nadir < 350 cells/µL may reflect a high chronic LPS stimulation *in vivo*, leading to a reduced capacity to respond to LPS. This is known as endotoxin tolerance, a mechanism with reduced cytokine response by immune cells to LPS after prior LPS exposure and have been described in patients with sepsis as well as in heathy persons after *in vivo* LPS administration (35–37). However, to our knowledge it is not established if increased microbial translocation in PWH leads to endotoxin tolerance, and we did not measure plasma LPS levels in our cohort. Also, in PWH with viral replication a smaller study reported a positive correlation between CD4+ T-cell count and R848-induced TNF-α and IL-12p40 in monocytes, consistent with our findings (38).

Importantly, participants with nadir CD4+ T-cell counts < 350 cells/µL had both lower LPS-, R848- and Poly I:C-induced IL-6 and IFN-γ concentrations than participants with higher nadir CD4+ T-cell counts reflecting an impairment of multiple TLR-signaling pathways in PWH with severe immunodeficiency. IFN-γ is an important mediator of the Th1 response towards intracellular pathogens and a decreased IFN-γ response increases the risk of intracellular infections with bacteria and parasites such as Mycobacterium Tuberculosis, Toxoplasma Gondii and lead to severe manifestations of viral infections (39). This is in line with infections observed in PWH progressing towards AIDS (40).

In our study, the induced innate immune response improved after 12 months of cART in the LPS-, R848- and Poly I:C-induced samples. The improvement after cART initiation is consistent with three smaller prospective studies, one using cryopreserved peripheral blood mononuclear cells stored pre- and post-cART reporting increased LPS-induced (TLR4) TNF-α response from monocytes after cART initiation (24), one using fresh PBMC reporting increased herpes simplex induced IFN-α concentration after 12 months of cART (22), and one using fresh PBMC reporting increased heat-inactivated influenza virus (TLR7)- and CpG oligodeoxynucleotide (TLR9)-induced IFN-α after 12 months of cART, albeit the concentrations did not reach the concentrations found in uninfected controls (23). Furthermore, initiation of cART is reported to dramatically reduce the risk of severe bacterial infection even in PWH with CD4+ T cell count > 500 cells/µL which may indicate a role for an improved innate response in line with our finding of improved LPS-induced responses (41).

The LPS- and R848-induced IFN-γ concentrations improved during cART and the same tendency was found for the Poly I:C induced IFN-γ concentration. Natural killer (NK)-cells are an important secretor of INF-γ and an altered phenotype of NK-cells with impaired function has been described in HIV infection which seems to be partly reversibly by cART (29, 42). Furthermore, upstream regulation of IFN-γ secretion may also contribute to increased IFN-γ response after cART initiation such as the secretion of the Th1 polarizing cytokine IL-12p40 produced by macrophages and dendritic cells. IL-12p40 concentrations were likewise improving after 12 months of cART in both the LPS- and Poly I:C-induced samples. Additionally, the loss of innate immune cells in PWH such as NK and dendritic cells and (partly) restoration after cART initiation could affect the innate response (29, 43). Intriguingly, only the induced IFN-γ concentration improved during cART in the R848-induced samples. Like R848, HIV as a single-stranded RNA virus, stimulates the TLR7/8 pathway and we could speculate that the HIV reservoir in innate immune cells, such as macrophages, contribute to a constant low-grade stimulation of this pathway (44).

In the Poly I:C-induced samples the IL-10 concentration increased 12 months after cART initiation. However, Poly I:C is not an inducer of IL-10 (26), and a positive correlation to the unstimulated IL-10 concentration indicate that the IL-10 concentration in the Poly I:C sample represents the systemic IL-10 concentration and that the increase 12 months after initiation of cART may reflect a systemic negative feed-back mechanism to the proinflammatory regeneration found to stimulation after 12 months of cART.

More participants had induced cytokine responses within the reference intervals after 12 months of cART. However, our data still suggest a substantial innate immune deficiency in PWH on cART compared with the majority of the participants still having LPS-induced IFN-α and IFN-γ and Poly I:C-iduced IL-6 and TNF-α concentrations below the reference intervals. This implies that residual innate immune dysfunction may contribute to the increased risk of both bacterial and viral infections in PWH on cART. However, longer follow-up time is needed to investigate if long-term cART can normalize the innate functional immune response.

The shift from highly positive intercorrelations of induced cytokines in the LPS- and R848-induced samples before cART to less and more diverse intercorrelations have been described in another study using the TruCulture^®^ technique in patients with hematological diseases undergoing allogeneic hematopoietic stem cell transplantation with subsequent immune reconstruction (45). However, the mechanism behind the different cytokine correlations before and after immune reconstruction remains unclear.

The inverse correlations between induced cytokine responses and viral load have been described in other studies using stimulation of TLR´s (17, 24). This finding implies that viral suppression contributes to improvement of the functional immune response, which is in line with the clinical observation that ongoing HIV replication is associated with increased risk of opportunistic diseases or death even in PWH with CD4+ T-cell count >350 cells/µL (46).

We did not find a pattern between the induced responses before cART initiation and the CD4+ T-cell recovery after 12 months of cART. It is possible that the variation in disease stages in our study population blunts the signal, but our analysis did not suggest a prognostic value of the TruCulture^®^ system in terms of CD4+ T-cell reconstruction in PWH.

The study is strengthened by its prospective design and the investigation of multiple TLR signaling pathways. Also, using a commercially available system, such as the TruCulture^®^ system makes it easily reproducible and enables comparison with other studies. A larger number of participants would have allowed for more sub-analyses within the different disease stages. However, our study population was homogeny with low hepatitis co-infection and generally reflects the variation in disease stages when initiating cART and allowed for analysis of the impact of CD4+ T-cells before treatment initiation. It is a limitation that we did not have data on the sex and age distribution in the reference group since both may impact the immune response (47). However, the same methods and laboratory were used to analyze samples from the reference group and the study group which is a strength. Also, we did not measure baseline plasma cytokine levels which could have provided additional information. The focus of this study was the innate immune response, but when using whole blood stimulation, a contribution from the other immunologically active circulating cells could occur. However, our ligands target TLR receptors that are predominantly expressed in innate immune cells, and with an incubation time of 22 hours we expect the measured immune response to reflect the capacity of the innate immune system, although some bystander stimulation of the adaptive immune system through released cytokines cannot be excluded. This was an exploratory study and results were not adjusted for multiple comparison which allows for the risk of type 1 error. However, a statistical analysis plan was predefined, and findings were interpreted in the context of the whole data set emphasizing signals across different stimuli.

In conclusion, HIV infection impaired the induced innate immune response and nadir CD4+ T-cell count might not only reflect T-cell deficiency but also impairment of the innate immune response. Even though an improvement was found after 12 months of cART, our data still suggest some residual innate immune deficiency. This could contribute to the increased susceptibility to infections found in PWH on cART. More extensive studies combining *in vitro* stimulation of multiple immunologic signaling pathways and the prevalence and type of infections in PWH with longer follow-up time are warranted to increase our knowledge of residual immune dysfunction in PWH and the risk of infections.

## Data availability statement

The dataset analyzed during the current study is available from the corresponding author on reasonable request.

## Ethics statement

The studies involving human participants were reviewed and approved by Committee on Health Research Ethics of the Capital Region of Denmark(H-17024315). The patients/participants provided their written informed consent to participate in this study.

## Author contributions

MH-S, JL, JG, SO, and SN designed the study. MH-S, DM, AH, SO, and SN were responsible for data collection. MH-S, SO, and SN were responsible for the statistical analyses. All authors interpreted the data. MH-S drafted the manuscript. All authors have critically revised and approved the final version.

## Funding

The study was funded by a research grant from Novo Nordisk Foundation, the Independent Research Fund (FSS), and Rigshospitalets Research Council. The funders had no role in design or in conducting the study.

## Conflict of interest

The authors declare that the research was conducted in the absence of any commercial or financial relationships that could be construed as a potential conflict of interest.

## Publisher’s note

All claims expressed in this article are solely those of the authors and do not necessarily represent those of their affiliated organizations, or those of the publisher, the editors and the reviewers. Any product that may be evaluated in this article, or claim that may be made by its manufacturer, is not guaranteed or endorsed by the publisher.
