# Supplementary material for: Improved induced innate immune response after cART initiation in people with HIV
Source: Front Immunol. 2022 Aug 17;13:974767. doi: 10.3389/fimmu.2022.974767 (PMC9428745; doi:10.3389/fimmu.2022.974767)
Supplement: Supplementary file 1 [file Table_1.docx]

**Supplementary**

|  | **LPS-induced** | | | | **R848-induced** | | | | **Poly I:C-induced** | | | |
| --- | --- | --- | --- | --- | --- | --- | --- | --- | --- | --- | --- | --- |
|  | % under reference interval | | % over reference interval | | % under reference interval | | % over reference interval | | % under reference interval | | % over reference interval | |
|  | Baseline | 12 months | Baseline | 12 months | Baseline | 12 months | Baseline | 12 months | Baseline | 12 months | Baseline | 12 months |
| **TNF-α** | 34 | 4 | 4 | 4 | 21 | 4 | 13 | 13 | 47 | 52 | 0 | 0 |
| **IL-1β** | 47 | 17 | 0 | 4 | 26 | 4 | 4 | 4 | 43 | 17 | 4 | 0 |
| **IL-6** | 30 | 0 | 9 | 13 | 17 | 0 | 13 | 0 | 69 | 56 | 0 | 0 |
| **IL-8** | 21 | 0 | 4 | 9 | 8 | 13 | 35 | 13 | 4 | 21 | 35 | 21 |
| **IL-10** | 34 | 13 | 9 | 17 | 26 | 15 | 0 | 4 | 0 | 0 | 13 | 13 |
| **IL-12p40** | 52 | 4 | 0 | 0 | 47 | 15 | 0 | 4 | 0 | 0 | 0 | 0 |
| **IL17A** | 56 | 21 | 0 | 35 | 47 | 43 | 9 | 13 | 0 | 0 | 4 | 4 |
| **IFN-α** | 68 | 65 | 0 | 0 | 53 | 26 | 0 | 4 | 53 | 21 |  | 9 |
| **IFN-γ** | 73 | 60 | 0 | 0 | 52 | 15 | 0 | 4 | 73 | 47 | 0 | 0 |

Caption: S1: Percentages of PWH with induced cytokine concentrations below and above reference values at baseline and after 12 months of cART

|  | **LPS** | | | **R848** | | | **Poly I:C** | | | **Unstimulated (NULL)** | | |
| --- | --- | --- | --- | --- | --- | --- | --- | --- | --- | --- | --- | --- |
|  | 4 weeks | 6 months | 12 months | 4 weeks | 6 months | 12 months | 4 weeks | 6 months | 12 months | 4 weeks | 6 months | 12 months |
| **TNF-α** | 0.205 | 0.074 | 0.012* | 0.279 | 0.215 | 0.062 | 0.608 | 0.418 | 0.340 | 0.532 | 0.009* | 0.056 |
| **IL-1β** | 0.186 | 0.015* | 0.008* | 0.297 | 0.229 | 0.203 | 0.612 | 0.187 | 0.059 | 0.261 | 0.993 | 0.693 |
| **IL-6** | 0.236 | 0.057 | 0.004* | 0.316 | 0.299 | 0.222 | 0.025* | 0.002* | 0.028* | 0.442 | 0.674 | 0.061 |
| **IL-8** | 0.110 | 0.388 | 0.012* | 0.466 | 0.006* | 0.002* | 0.682 | 0.037* | 0.012* | 0.797 | 0.039* | 0.002* |
| **IL-10** | 0.678 | 0.222 | 0.228 | 0.069 | 0.534 | 0.616 | 0.429 | 0.139 | 0.028* | 0.323 | 0.426 | 0.759 |
| **IL-12p40** | 0.137 | 0.099 | 0.002* | 0.145 | 0.085 | 0.052 | 0.005* | 0.051 | 0.039* | 0.194 | 0.274 | 0.678 |
| **IL-17A** | 0.118 | 0.826 | 0.029* | 0.172 | 0.878 | 0.237 | 0.470 | 0.106 | 0.100 | 0.557 | 0.817 | 0.167 |
| **IFN-α** | 0.911 | 0.931 | 0.838 | 0.070 | 0.423 | 0.061 | 0.033* | 0.050* | 0.017* | 0.234 | 0.726 | 0.988 |
| **IFN-γ** | 0.002* | 0.001* | 0.006* | 0.007* | 0.004* | 0.004* | 0.437 | 0.169 | 0.077 | 0.159 | 0.010* | 0.676 |

Caption: S2: P-values for the cytokine concentration during the first year of cART compared to baseline values. Evaluated with linear mixed models. Please see details in the statistics section.


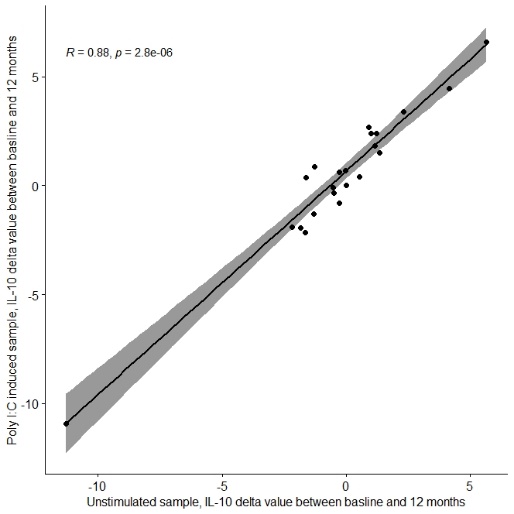

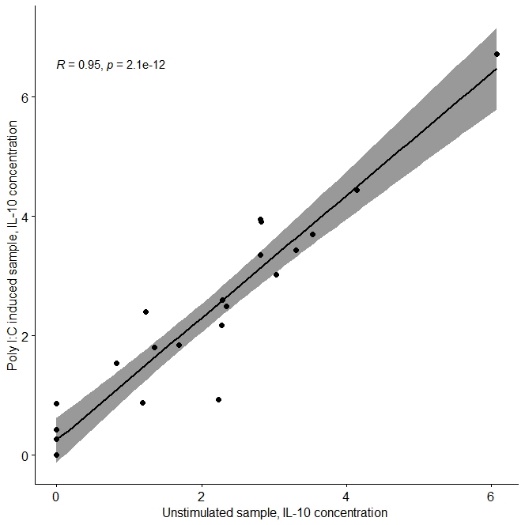


Caption: S3: A: Correlation between IL-10 concentrations in the unstimulated and the Poly I:C induced sample 12 months after cART initiation. B: Correlation between IL-10 delta value from baseline to 12 months after cART initiation in the unstimulated and the Poly I:C induced sample.

|  | LPS | | R848 | | Poly I:C | |
| --- | --- | --- | --- | --- | --- | --- |
|  | Rho | p-value | Rho | p-value | Rho | p-value |
| TNF-α | -0.134 | 0.434 | -0.199 | 0.328 | -0.009 | 0.966 |
| IL-1β | -0.114 | 0.580 | -0.265 | 0.190 | 0.090 | 0.664 |
| IL-6 | -0.153 | 0.457 | -0.077 | 0.708 | -0.119 | 0.562 |
| IL-8 | 0.136 | 0.508 | 0.680 | 0.086 | -0.007 | 0.974 |
| IL-10 | -0.135 | 0.510 | -0.158 | 0.440 | 0.143 | 0.484 |
| IL-12p40 | 0.026 | 0.899 | -0.047 | 0.819 | 0.227 | 0.266 |
| IL17A | 0.142 | 0.488 | 0.081 | 0.692 | 0.399 | 0.044* |
| IFN-γ | 0.019 | 0.929 | -0.204 | 0.316 | 0.068 | 0.739 |

Caption: S4: Spearman correlations between baseline induced cytokine concentrations and delta CD4+ T-cell increase 12 months after cART initiation.
